# Symbionts Commonly Provide Broad Spectrum Resistance to Viruses in Insects: A Comparative Analysis of *Wolbachia* Strains

**DOI:** 10.1371/journal.ppat.1004369

**Published:** 2014-09-18

**Authors:** Julien Martinez, Ben Longdon, Simone Bauer, Yuk-Sang Chan, Wolfgang J. Miller, Kostas Bourtzis, Luis Teixeira, Francis M. Jiggins

**Affiliations:** 1 Department of Genetics, University of Cambridge, Cambridge, United Kingdom; 2 Laboratories of Genome Dynamics, Department Cell and Developmental Biology, Center of Anatomy and Cell Biology, Medical University of Vienna, Vienna, Austria; 3 Department of Environmental and Natural Resources Management, University of Patras, Agrinio, Greece; 4 Biomedical Sciences Research Center “Alexander Fleming”, Vari, Greece; 5 Insect Pest Control Laboratory, Joint FAO/IAEA Division of Nuclear Techniques in Food and Agriculture, Vienna, Austria; 6 Instituto Gulbenkian de Ciência, Oeiras, Portugal; Stanford University, United States of America

## Abstract

In the last decade, bacterial symbionts have been shown to play an important role in protecting hosts against pathogens. *Wolbachia*, a widespread symbiont in arthropods, can protect *Drosophila* and mosquito species against viral infections. We have investigated antiviral protection in 19 *Wolbachia* strains originating from 16 *Drosophila* species after transfer into the same genotype of *Drosophila simulans*. We found that approximately half of the strains protected against two RNA viruses. Given that 40% of terrestrial arthropod species are estimated to harbour *Wolbachia*, as many as a fifth of all arthropods species may benefit from *Wolbachia*-mediated protection. The level of protection against two distantly related RNA viruses – DCV and FHV – was strongly genetically correlated, which suggests that there is a single mechanism of protection with broad specificity. Furthermore, *Wolbachia* is making flies resistant to viruses, as increases in survival can be largely explained by reductions in viral titer. Variation in the level of antiviral protection provided by different *Wolbachia* strains is strongly genetically correlated to the density of the bacteria strains in host tissues. We found no support for two previously proposed mechanisms of *Wolbachia-*mediated protection — activation of the immune system and upregulation of the methyltransferase *Dnmt2*. The large variation in *Wolbachia*'s antiviral properties highlights the need to carefully select *Wolbachia* strains introduced into mosquito populations to prevent the transmission of arboviruses.

## Author Summary

In recent years it has been discovered that many organisms are infected with bacterial symbionts that protect them against pathogens. *Wolbachia* is a bacterial symbiont that is found in many species of insects, and several strains are known to protect the insects against viral infection. We took 19 strains of *Wolbachia* from different species of *Drosophila* fruit flies, transferred them into *Drosophila simulans*, and then infected these flies with two different viruses. We found that about half of the strains slowed the death of flies after viral infection. Given that 40% of terrestrial arthropods may be infected with *Wolbachia*, this suggests that many species may benefit from this protection. These increases in survival were tightly linked to reductions in the levels of the virus in the insect, suggesting that *Wolbachia* is reducing the viruses' ability to replicate. Despite the two viruses we used being very different, the level of protection that a *Wolbachia* strain provided against the two viruses tended to be very similar, suggesting that a single general mechanism underlies the antiviral effects. The extent to which a *Wolbachia* strain provides protection against viral infection depends largely on the bacterial density— the more *Wolbachia*, the greater the protection.

## Introduction

Heritable symbionts are major players in arthropod evolution owing to their high incidence and the diversity of effects they have on their host's phenotype [1]–[3]. Primary (obligate) symbionts are mutualists that play some essential role — typically synthesizing nutrients missing from the insect's diet — and they often form stable associations with their hosts that can last for many millions of years [4]–[6]. Secondary (facultative) symbionts have more diverse effects, which range from parasitism to mutualism [3], [7]. The parasites mostly manipulate their host's reproduction to enhance their transmission to the next generation, for example by distorting the sex ratio towards females (the sex that transmits the bacteria). The mutualists can supply nutrients, or protect against environmental stresses [8] or natural enemies [9]–[11]. Furthermore, symbionts can combine several strategies at once, with some ‘Jekyll and Hyde’ strains simultaneously exhibiting mutualistic and parasitic phenotypes [12].

As secondary symbionts occasionally jump between different host species [13]–[16], they can result in rapid evolutionary change in their hosts. This process may be quite different to selection acting on the host genome, as when a host acquires a novel symbiont it can instantly acquire a complex adaptation encoded by many genes. Striking examples of rapid evolution resulting from the spread of symbionts include a *Rickettsia* bacterium infecting whiteflies which rapidly spread through US populations by causing sex ratio distortion as well as increased fecundity and survival [17], and a *Spiroplasma* bacterium that spread through populations of *Drosophila neotestacea*, protecting the hosts against a parasitic nematode [10].

*Wolbachia* is a maternally-transmitted alphaproteobacterium that is estimated to infect around 40% of terrestrial arthropods [18] and can act as both a parasite and a mutualist. Until recently it was viewed primarily as a parasite that manipulates host reproduction, most commonly by inducing cytoplasmic incompatibility (CI) [19]–[23]. CI allows *Wolbachia* to invade populations by causing embryonic mortality when uninfected females mate with infected males, thus conferring a selective advantage to infected females [24]. Recently it was discovered that *Wolbachia* can also protect *Drosophila melanogaster* against several RNA viruses [25], [26], and subsequently similar antiviral effects have been reported in other *Drosophila* species [27], [28], as well as in mosquitoes [29]–[32]. In most cases, *Wolbachia* has been shown to be associated with a decrease in viral titer [26], [27], [29]. However, *Wolbachia* increased the survival of the flies but had no effect on viral titer in *D. melanogaster* infected with Flock House virus (FHV) [26] as well as in one case in *D. simulans* infected with *Drosophila* C virus (DCV) [27], suggesting that *Wolbachia* might also allow its host to tolerate viral infections without affecting the pathogen load. *Wolbachia* has also been associated with protection against filarial nematodes, *Plasmodium* parasites and pathogenic bacteria in mosquitoes [29], [33]–[36]. However, it is not known whether the mechanisms of protection acting against these parasites are the same that are involved in protection against viruses.

Antiviral protection by *Wolbachia* could potentially be used to control vector-borne diseases such as dengue fever [37], [38]. When artificially introduced into *Aedes aegypti*, the main vector of dengue virus, *Wolbachia* was shown to limit the replication of dengue virus as well as chikungunya, yellow fever and West Nile viruses [29], [39], [40]. Furthermore, when *Wolbachia* infected mosquitoes were released into the wild, the bacterium spread through the mosquito populations due to the induction of CI [41], [42].

In both *Drosophila* and mosquitoes, different *Wolbachia* strains are associated with different levels of antiviral protection [27], [32], [39], [43], [44], even among very closely related strains [45]. The causes of this variation are not entirely clear, as relatively few *Wolbachia* strains have been characterized for their level of protection, not all studies control the host genetic background, and none have controlled for the confounding effects of the bacterial phylogeny. Nonetheless, several studies found that the *Wolbachia* strains with the highest density within the host provide the strongest protection against viruses, and tissue tropism may also play a role [27], [30], [44]–[47]. Overall, little is known about how commonly *Wolbachia* protects insects against viral infection, how this trait is distributed across the *Wolbachia* phylogeny, and therefore to what extent it has contributed to the evolutionary success of *Wolbachia*.

The mechanisms by which *Wolbachia* protects hosts against viruses remain to be elucidated. The protection could be caused by direct interactions between *Wolbachia* and viruses, competition for shared resources, or indirectly through the regulation of host gene expression [26]. In particular, it was speculated that *Wolbachia* infection may up-regulate the host immune system. While this was shown to occur after transinfection of *Wolbachia* from *Drosophila* into *Ae. aegypti* and *Anopheles gambiae* [33], [48]–[50], such an effect was not observed in *D. melanogaster* naturally infected with *Wolbachia* [50]–[55] or transinfected with a non-native strain [44]. Similarly, the small interfering RNA (siRNA) pathway, which provides broad-spectrum antiviral defense in insects, is not required for *Wolbachia* to confer antiviral protection in flies [56]. Recent results suggest that, in *Ae. aegypti*, *Wolbachia* has an indirect effect on viral replication through the manipulation of host microRNAs [57], [58]. In this species *Wolbachia* suppresses the expression of *AaDnmt2*, a methyltransferase gene, by up-regulating the microRNA aae-miR-2940 [57]. Overexpression of *AaDnmt2* decreases *Wolbachia* density and increases the titer of Dengue virus, suggesting a causal link between *Wolbachia* and viral replication. However, *Dnmt2*, the homolog of *AaDnmt2* found in *D. melanogaster*, was shown to have an antiviral effect against *Drosophila* C virus [59], contradicting the effect observed in case of dengue infection.

To overcome the lack of experimental tools available for *Wolbachia* — the bacterium cannot be cultured outside of insect cells and cannot be genetically manipulated or cloned — we have taken a comparative approach, looking for genetic correlations between levels of antiviral protection and potential causes such as changes in gene expression. To allow us to do this, we compared the level of protection of 19 *Wolbachia* strains from a diverse range of *Drosophila* species that we transferred into a common *D. simulans* genetic background. We used *Drosophila* C virus and Flock House virus, which are both RNA viruses with positive-sense single-stranded genomes. *Drosophila* C virus belongs to the family *Dicitroviridae* [60] and is naturally found in *D. melanogaster* and *D. simulans* [61]–[63]. FHV belongs to the family *Nodaviridae* and was initially isolated from a beetle [64].

Using this comparative approach, we show that *Wolbachia* strains vary considerably in the extent to which they increase the survival of flies after viral infection. There is little specificity, with strong genetic correlations between protection against FHV and DCV, despite these viruses being distantly related. The increases in survival can largely be explained by *Wolbachia* reducing viral titer. The variation in antiviral protection is largely explained by differences in the density of *Wolbachia* in host tissues. However, there is no evidence that either activation of the humoral immune response or up-regulation of the methyltransferase gene *Dnmt2* play any role in antiviral protection.

## Results

### Diversity of *Wolbachia* in the genus *Drosophila*

**Figure 1:**
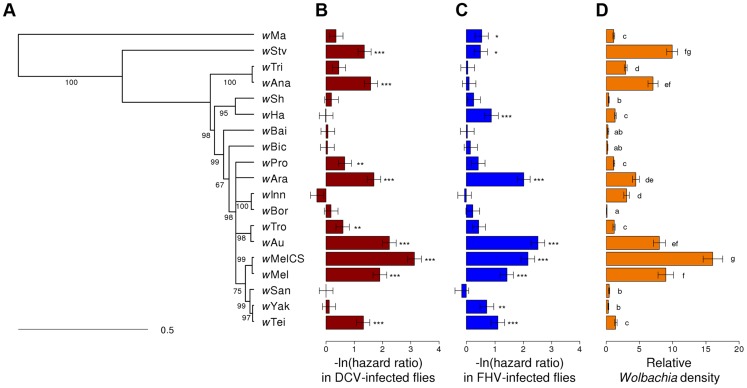
Phylogeny of *Wolbachia* strains and respective level of protection and within-host density. (A) The phylogeny is based on the sequence of the MLST genes *16S rRNA*, *aspC*, *atpD*, *ftsZ*, *sucB*, *groEL*, *coxA* and *fbpA*. Branch labels represent posterior support values. Nodes with less than 50% support were collapsed. The scale bar indicates time in coalescent units. (B–C) Flies were either infected with (B) DCV or (C) FHV. Survival is expressed as the negative natural log of the hazard ratio compared to *Wolbachia-*free flies, as estimated from a Cox's mixed-effect model. Error bars are standard errors. Symbols above the bars give the significance relative to the *Wolbachia*-free controls (*: *P*<0.05; **: *P*<0.01; ***: *P*<0.001). (D) *Wolbachia* density is expressed as the ratio of *Wolbachia* genomic DNA to *Drosophila* genomic DNA, as estimated by quantitative PCR. Different letters indicate significant differences based on a Tukey's honest significance test on ln-transformed data.

We assembled a panel of 19 *Wolbachia* strains that naturally infect 16 different species of *Drosophila* (**Table 1**). We reconstructed the phylogeny of these strains using sequences from eight multilocus sequence typing (MLST) genes and a Bayesian method that accounts for recombination between strains [65]. The phylogeny reveals that 18 of the strains clustered in what is commonly regarded as the supergroup A [66], with *w*Ma being the only strain from the supergroup B (Figure 1A). Many of the strains are very closely related.

**Table 1:** *Wolbachia* strains used in this study.

*Wolbachia* strain	Original Host Species	*Wolbachia* Supergroup	*Drosophila* Lines
*w*Ana^a^^,^ c	*D. ananassae* g	A	14024-0371.11^m^
*w*Ara^a^^,^ c	*D. arawakana* g	A	15182-2260.00^m^
*w*Bai^a^^,^ c	*D. baimaii* g	A	14028-0481.01^m^
*w*Bic^a^^,^ c	*D. bicornuta* g	A	14028-0511.00^m^
*w*Bor^a^^,^ c	*D. borealis* h	A	PG05.16^m^
*w*Inn^a^^,^ d	*D. innubila* i	A	KB183^l^
*w*MelCS^a^^,^ c	*D. melanogaster* j	A	DrosDel *w^1118^ iso* m
*w*Mel^a^^,^ e	*D. melanogaster* e	A	KB179^l^, KB101^m^
*w*Pro^a^^,^ c	*D. prosaltans* k	A	WM0019^m^
*w*San^a^^,^ f	*D. santomea* f	A	KB161^l^, STO.9^m^
*w*Sh^a^^,^ c	*D. sechellia* g	A	14021-0248.08^m^
*w*Ma^b^^,^ f	*D. simulans* f	B	KB176^l^, KB153^m^
*w*Ha^b^^,^ f	*D. simulans* f	A	KB178^l^, KB29^m^
*w*Au^b^^,^ f	*D. simulans* f	A	KB177^l^, KB30^m^
*w*Stv^a^^,^ c	*D. sturtevanti* g	A	14043-0871.10^m^
*w*Tei^a^^,^ f	*D. teissieri* f	A	KB156^l^, 0257.0^m^
*w*Tri^a^^,^ c	*D. triauraria* g	A	14028-0651.00^m^
*w*Tro^a^^,^ c	*D. tropicalis* g	A	14030-0801.0^m^
*w*Yak^a^^,^ f	*D. yakuba* f	A	KB165^l^, SA3^m^
uninfected^e^^,^ f	*-*	-	KB171^l^

The *Wolbachia* strains were traninfected into *D. simulans* STCP either by

a by microinjection or

b introgression. The transinfection was done.

c during this study, or previously by

d [83],

e [82]or

f [23]. The *Wolbachia* strains were obtained from and/or described by

g [97] (San Diego Drosophila Species Stock Center),

h [98],

i [21],

j [45] and

k Wolfgang Miller (unpublished). The fly line names either refer to

l the transinfected *D. simulans* stock or

m the original host.

To allow us to compare the level of antiviral protection that the different *Wolbachia* strains provide to their hosts, we transferred them into the same inbred *D. simulans* genetic background. Eleven of the strains were transferred as part of this study, and the remaining eight have been reported before (**Table 1**).

### *Wolbachia* strains vary in the extent to which they increase survival after viral infection

**Figure 2:**
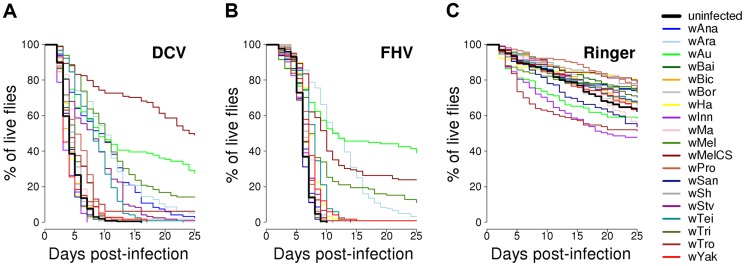
Survival of flies carrying different *Wolbachia* strains or being *Wolbachia*-free. Flies were either infected with (A) DCV, (B) FHV or (C) mock-infected with Ringer's solution.

Flies from the 19 *D. simulans* lines carrying the different *Wolbachia* strains, together with a *Wolbachia-*free control, were stabbed with a needle that had been dipped in DCV, FHV or Ringer's solution (2528, 2527 and 2492 flies were stabbed for each treatment respectively). We then followed their survival for 25 days (Figure 2A–C). In the mock-infected flies (Ringer), there was significant heterogeneity among the 20 fly lines (Cox's mixed-effect model, χ^2^ = 47, d.f. = 19, *P* = 0.0004; Figure S1), possibly reflecting either intrinsic effects of *Wolbachia* on survival or some other difference between the lines, such as remaining differences in the host genetic background. The overall survival of the mock-infected flies was low, likely due to this being a weak inbred stock (Figure 2C).

There was a substantial variation among *Wolbachia* strains in the degree to which they protect their hosts against viral infection (Figure 2A & B). Twelve of the *Wolbachia-*infected lines showed significantly reduced mortality relative to the *Wolbachia*-free flies within either the DCV or FHV treatments (Figure 1B & 1C). To account for the slight variation in the survival of the mock-infected controls, we tested whether survival of the 20 fly lines was affected by a statistical interaction between the *Wolbachia* strain and infection (whether the flies were infected with a virus or mock-infected). There was a highly significant interaction for both DCV and FHV (Cox's mixed-effect models; DCV: χ^2^ = 127.4, d.f. = 19, *P*<10^−15^; FHV: χ^2^ = 107.6, d.f. = 19, *P*<10^−14^). Using this more conservative approach of testing for an interaction of *Wolbachia* and infection treatment, nine of the 19 *Wolbachia* strains provided a significant level of antiviral protection, with six protecting against both viruses, one protecting against just FHV and two protecting against just DCV (Table S1). This protective phenotype is widespread across the *Wolbachia* phylogeny and is not restricted to particular clades (Figure 1A–C).

### There is a strong genetic correlation in the level of protection against DCV and FHV

**Figure 3:**
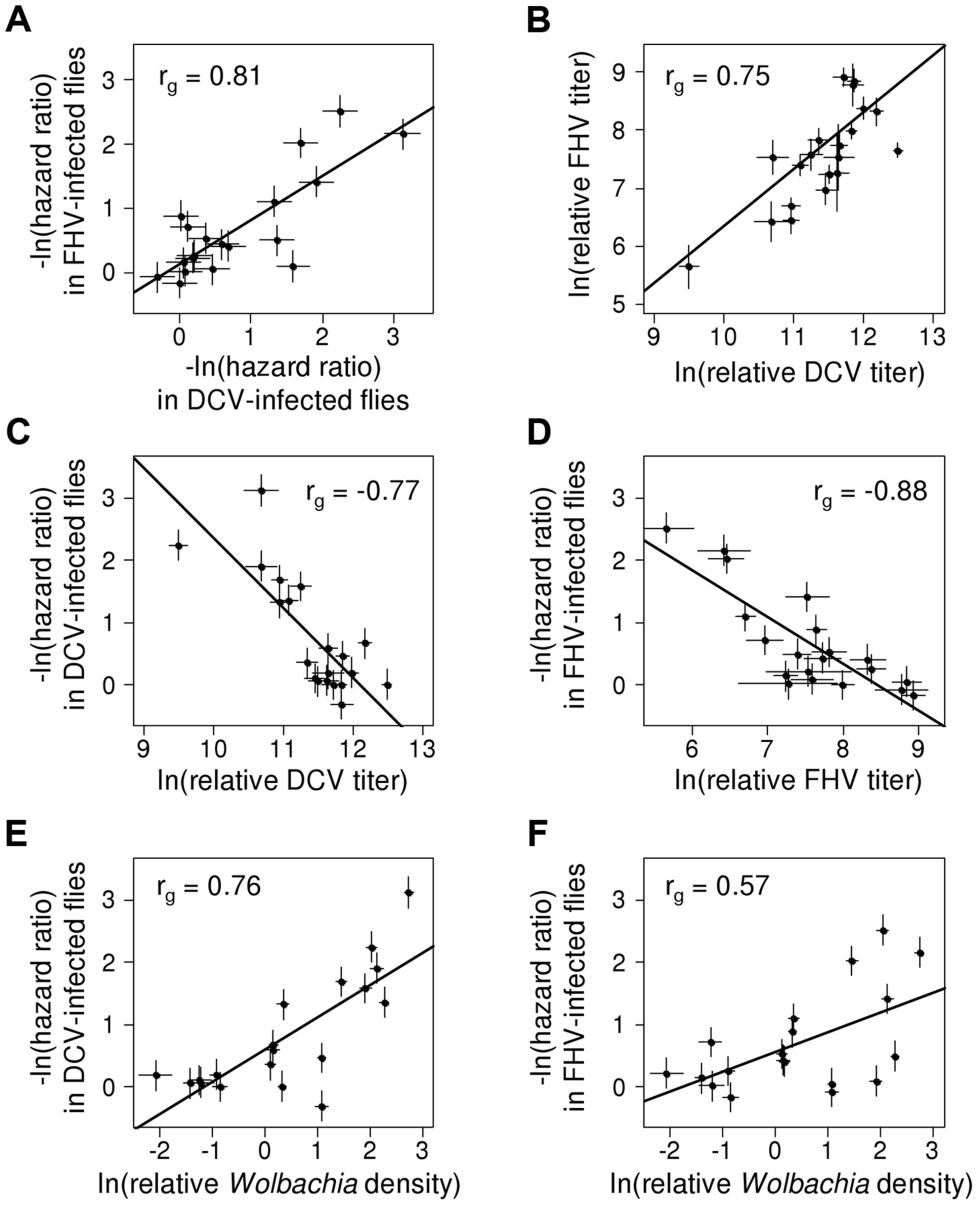
Correlation between protection, viral titers and *Wolbachia* density. Dots indicate mean value of the traits for each *Wolbachia* strain. Error bars are standard errors. Solid lines show predicted values from linear regressions for illustrative purposes. *r_g_* is the genetic correlation between traits. (A) Correlation of survival between DCV- and FHV-infected flies (negative natural log of hazard ratios). (B) Correlation between DCV and FHV titers. (C–D) Correlation between viral titer and survival following (C) DCV infection or (D) FHV infection. Viral titers were estimated as viral RNA concentrations relative to the *Drosophila* gene *EF1α100E*. (E–F) Relationship between *Wolbachia* density and survival in (E) DCV- and (F) FHV-infected flies. *Wolbachia* density was estimated as the ratio between copy numbers ofthe Wolbachia gene *atpD* and the *Drosophila* gene *Actin 5C*.

To examine the extent to which the effects of *Wolbachia* are specific to different viruses, we estimated the genetic correlation between protection to DCV and FHV (the proportion of the genetic variance shared by the two traits). The genetic correlation between protection to DCV and FHV was high (Model 1: *r_g_* = 0.81, 95% CI: 0.55,0.97; Figure 3A), indicating that most of the genetic variance in antiviral protection affects both DCV and FHV. There was no evidence of a genetic correlation between the survival of virus-infected and mock-infected flies (Model 1, DCV-Ringer: *r_g_* = 0.31, 95% CI = −0.37,0.87; Model 1, FHV-Ringer: *r_g_* = 0.61, 95% CI = −0.14,0.99).

This genetic correlation between viruses could arise either because there is a causal relationship between DCV and FHV protection, or as a consequence of common ancestry (phylogenetic non-independence). We used a phylogenetic mixed model to partition the variance in the two traits into a component that can be explained by correlations across the *Wolbachia* phylogeny and a strain specific component that is independent of phylogeny. If there is a causal link between the traits, then the strength of their association will be the same for the phylogenetic and strain components. There was no significant difference in the strength of the genetic correlation for the phylogenetic and strain-specific components, consistent with a causal link between the traits. As we have limited power to separate these effects in a single model, we also fitted a model with just the phylogenetic effect. This model again produced a very similar correlation between viruses (*r_g_* = 0.95, 95% CI: 0.84,0.99), and the convergence of parameter estimates was improved.

### Increased survival is genetically correlated to reduced viral titers

**Figure 4:**
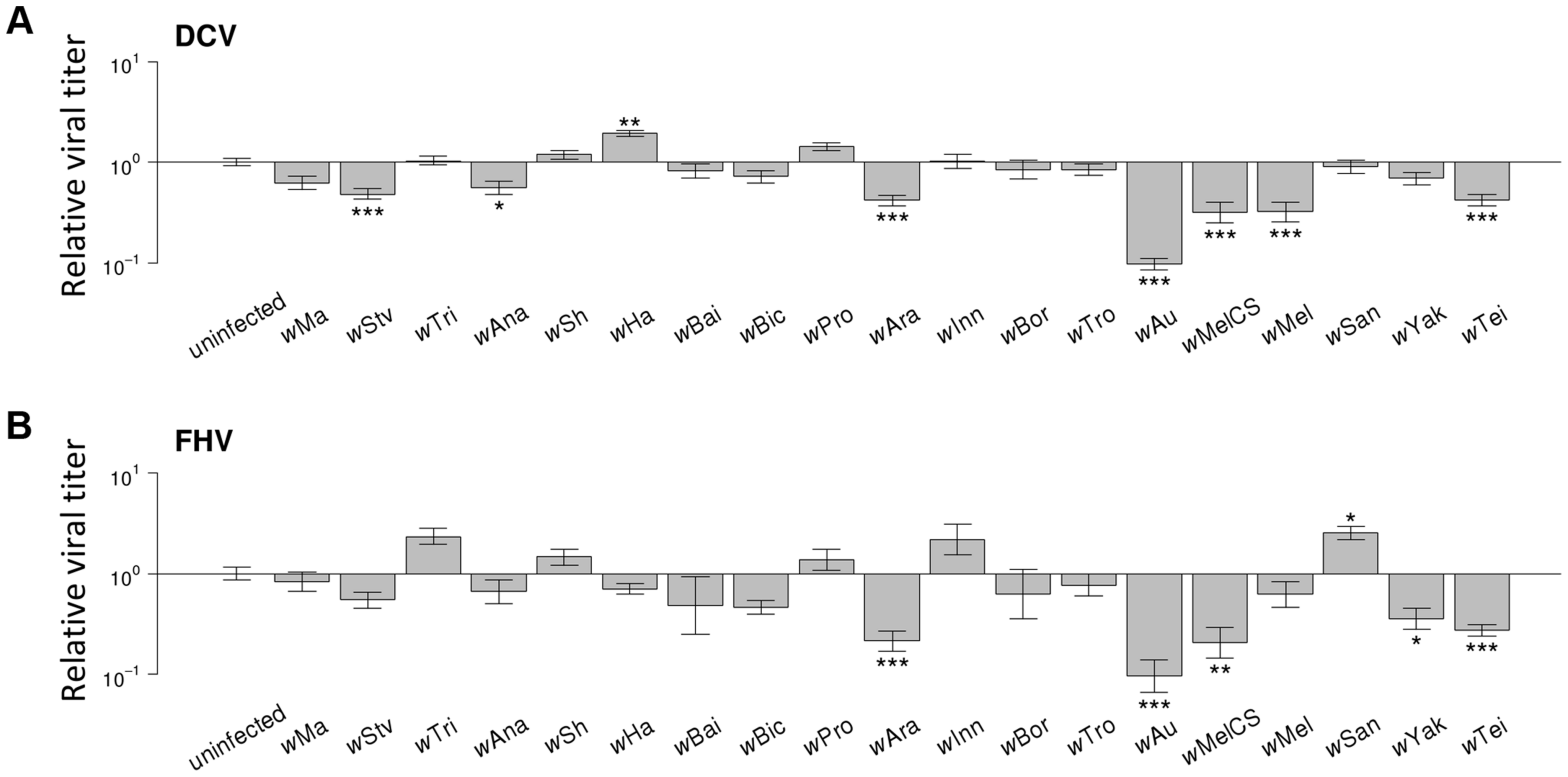
Effect of *Wolbachia* strains on viral titers. (A–B) Relative viral titer in (A) DCV- and (B) FHV-infected flies. Relative titers are normalised by the mean titer of *Wolbachia*-free controls (uninfected). Error bars are standard errors. Symbols above the bars give the significance relative to the *Wolbachia*-free controls based on a Dunnett's test (*: *P*<0.05; **: *P*<0.01; ***: *P*<0.001).

To investigate the effect of *Wolbachia* on viral titers, flies were stabbed with DCV or FHV, and relative viral RNA levels measured at two days post infection (dpi) by reverse transcription-quantitative PCR (RT-qPCR). For both DCV and FHV, viral titer was affected by the *Wolbachia*-infection status of flies (ANOVA on ln(viral titer); DCV: *F_19,165_* = 23.4, *P*<10^−16^; FHV: *F_19,166_* = 12.1, *P*<10^−16^; Figure 4A & 4B). *Wolbachia* strains tended to have similar effects on the two viruses, with a strong positive genetic correlation across the *Wolbachia* strains between titers of DCV and FHV (Model 2: *r_g_* = 0.75, 95% CI = 0.48,0.94; Figure 3B).

*Wolbachia* could increase the survival of *Drosophila* after infection either by reducing viral titers (increasing resistance), or by allowing flies to better cope with infection damage (increasing tolerance). To test whether *Wolbachia* provides resistance to infection, we compared the survival of flies and viral titers across the 19 *Wolbachia* strains. Relative to the *Wolbachia-*free control, seven of the strains were individually associated with significantly reduced DCV titers, and five with reduced FHV titers (Figure 4A & 4B). Interestingly, for the strains *w*Ha in DCV- and *w*San in FHV-infected flies, there was a significant increase in viral RNA levels (Figure 4A & 4B) and this result was replicable by repeating the experiment on these lines (J. Martinez, personal observation). Overall, we found that the titer of both DCV and FHV was negatively genetically correlated to the survival of DCV and FHV infected flies respectively (Model 3, DCV: *r_g_* = −0.77, 95% CI = −0.95,−0.49; Model 4, FHV: *r_g_* = −0.88, 95% CI = −1.00,−0.64; Figure 3C & 3D). Therefore, resistance could be the primary explanation for *Wolbachia-*induced protection.

To understand how *Wolbachia* affects the dynamics of infection, we followed DCV and FHV titers for five days in *Wolbachia*-free flies and flies infected with the protective strain *w*Au that conferred strong protection against both viruses, the non-protective strain *w*Sh and *w*Ana, which protected against DCV but not FHV. In all treatments, including in the presence of the strongly protective strain *w*Au, DCV and FHV were able to replicate within the flies (Figure S2). For example, at 2 dpi, DCV titres had increased ∼18.000-fold in *w*Au-infected flies compared to ∼290.000-fold in *Wolbachia*-free flies. At 2 dpi, the timepoint chosen in the previous experiment, viruses were still in their growth phase, with a plateau of DCV titres being reached at around 3 dpi (Figure S2). Resistance conferred by *w*Au seemed to occur earlier against FHV (1 dpi at start growth phase) than against DCV (2 dpi at end growth phase) and viral titers were reduced from those points on, including at the end of the growth phase.

### *Wolbachia* density is positively genetically correlated to antiviral protection

There was significant variation in the relative density of the different *Wolbachia* strains (ANOVA on ln(relative density): *F_18,150_* = 115.6, *P*<10^−16^; Figure 1D) in 3–6 day old virus-free flies (the same age as the flies that were infected to estimate survival and viral titer). The highest density strain, *w*MelCS, had 114 fold higher density than the lowest density strain, *w*Bor.

Flies infected with high density *Wolbachia* strains tended to live longer after viral infection (Figure 3E & F) and had lower viral titers. This is reflected in strong genetic correlations between bacterial density and survival after both DCV and FHV infection (Model 1; DCV: *r_g_* = 0.76, 95% CI: 0.49,0.93; FHV: *r_g_* = 0.57, 95% CI: 0.20,0.86). This appears to be a specific effect of *Wolbachia* on survival after viral infection as there is no support for a correlation between *Wolbachia* density and the survival of the mock-infected flies in this assay (Model 1; Ringer: *r_g_* = 0.18, 95% CI: −0.41,0.77). Similar to the survival analysis, bacterial density is negatively genetically correlated with DCV titers (Model 2: *r_g_* = −0.53, 95% CI: −0.82,−0.13). However, there is no support for a correlation between FHV titer and *Wolbachia* density (Model 2: *r_g_* = −0.29, 95% CI: −0.65,0.18).

To examine whether there is likely to be a causal relationship between *Wolbachia* density and survival after viral infection, we partitioned the variance in these traits into components that are dependent and independent of the *Wolbachia* phylogeny. The regression coefficient of survival against bacterial density was not significantly different for the phylogenetic and strain components for the two viruses. We are therefore unable to find evidence to suggest that this correlation is an artefact of phylogenetic relatedness, although we would caution that this analysis has very limited statistical power (non-significant phylogenetic component). Given this limited power to partition the variance across the two components, we also fitted a model with phylogenetic component only, and again the correlations between protection and *Wolbachia* density were similar to the model without the phylogeny (DCV: *r_g_* = 0.71, 95% CI: 0.32,0.92; FHV: *r_g_* = 0.63, 95% CI: 0.24,0.90).

### Antiviral protection is not correlated with immune or methyltransferase gene expression

We finally investigated if antiviral protection could be explained by an effect of *Wolbachia* on host gene expression. We first tested the hypothesis that *Wolbachia* prime the immune system of flies, by measuring the expression of *Drosomycin* and *Diptericin* as reporters of Toll and IMD pathway activation respectively. We did not detect a significant genetic correlation between expression of either immune genes and DCV protection (Model 5; *Drosomycin*: *r_g_* = 0.26, 95% CI: −0.37,0.83; *Diptericin*: *r_g_* = −0.05, 95% CI: −0.79,0.66; Figure S3A & S3C) or FHV protection (Model 6; *Drosomycin*: *r_g_* = 0.25, 95% CI: −0.29,0.72; *Diptericin*: *r_g_* = 0.28, 95% CI: −0.37,0.95; Figure S3B & S3D). Finally, the expression of a putative candidate for protection, the methyltransferase gene *Dnmt2*, was not significantly affected by the *Wolbachia*-infection status (ANOVA on ln(expression level); DCV: *F_16,122_* = 1.56, 0.09; FHV: *F_16,122_* = 0.85, *P* = 0.63; Figure S3E & S3F) and did not show any correlation with level of protection in DCV-infected (Model 5; *Dnmt2*: *r_g_* = 0.12, 95% CI: −0.59,0.90) or FHV-infected flies (Model 6; *Dnmt2*: *r_g_* = −0.35, 95% CI: −0.98,0.49).

## Discussion

Protective symbionts can be an important component of an organism's defenses against infection, in some cases even being the primary mode of defense [67]. Despite their importance being increasingly recognized, studying these symbionts remains challenging. Many cannot survive outside of host cells, be genetically manipulated or cloned. Our approach to circumvent these problems has been to assemble a large panel of different *Wolbachia* strains in a common host genetic background. This allows us to detect genetic correlations between traits, and infer whether these traits are causally linked.

Our results suggest that symbionts may play a role in protecting a substantial proportion of insect species against viral infection. *Wolbachia* is probably the most widespread symbiont in arthropods [68] and its wide distribution is partly attributable to its ability to manipulate host reproduction as well as its tendency to be horizontally-acquired between different host species over evolutionary time scales [2]. Recently, it has been shown to confer protection against natural enemies, in particular against RNA viruses [25], [26], [29]. By assessing the level of protection among several *Wolbachia* strains, we showed that, far from being an exception, *Wolbachia*-mediated protection is a common phenomenon, which could potentially have contributed to its evolutionary success.

Among the tested strains, about half were able to confer some level of protection in *D. simulans*. Assuming that *Wolbachia* is found in 40% of arthropod species [18], our results suggest that 20% of arthropods may benefit from such a protection. This extrapolation relies on strains retaining their ability to protect their original host, but the host species could also influence the expression of the protective phenotype. It was previously shown that protective strains native to *D. melanogaster* also protect mosquito hosts after artificial transfer. In contrast, the strain *w*Inn, which did not confer protection in this study, was previously found to protect against FHV in its original host *D. innubila* [28]. Host genotype effects on the *Wolbachia* density have previously been found [69], [70]. Given the correlation between protection and density, the expression of protection is likely to be under the control of both the *Wolbachia* strain and the host genotype.

*Wolbachia* has previously been shown to protect insects against a remarkably taxonomically diverse array of RNA viruses [25], [26], [29], [31], [40]; and this could either reflect a broad-spectrum antiviral mechanism or *Wolbachia* may have independently evolved different ways of targeting different viruses. Previously it has been observed that strains that protect strongly against one virus tend to protect against other viruses, suggesting the former explanation is true [27], [44]–[45]. We found that an estimated 81% (*r*_g_ = 0.81) of the genetic variation among strains in DCV and FHV protection is common to the two viruses. Furthermore, this pattern appears to be independent of the bacterial phylogeny, indicating that the same genes underlie the level of protection to the two viruses tested. This supports the hypothesis that *Wolbachia* has a single broad-spectrum mechanism of antiviral protection.

The increased survival of *Wolbachia*-infected flies after viral infection could result from the symbiont increasing either resistance or tolerance to infection [71], [72]. Resistance occurs where increases in survival are caused by reductions in viral titers, while tolerance describes the situation where hosts are better able to survive a given viral load [71]. Both of these effects have been ascribed to the antiviral properties of *Wolbachia* in the past [26], [27], [29], [31], [40], [44], [45]. Our analysis allows us to test the effect of resistance by estimating the proportion of the variation in survival that can be explained by differences in viral titer. The genetic correlation between titer and survival was very high for both viruses, so in this instance it seems likely that the between-strain variation in survival is mainly due to resistance to virus infection. Our data cannot exclude a role for tolerance, as *Wolbachia* may be altering the amount of harm that a given viral titre causes. However, were this to be the case, then it is likely to be a common underlying link, such as both traits relying on *Wolbachia* density or the same mechanism.

In some studies it was shown that *Wolbachia* infection can lead to higher viral titers or virus-induced mortality [73], [74]. Interestingly, in our experiment two *Wolbachia* strains were associated with an increase in viral titer, although not with increased mortality. This is a tantalizing result, which would suggest care should be taken when introducing *Wolbachia* into disease vector populations. However, we would caution that this result needs to be investigated in more detail – we measured many traits across many strains, so rare outliers could be an artefact of confounding factors like remaining differences in the genetic background of the strains.

The density of *Wolbachia* plays a key role in determining the level of antiviral protection it provides to its host. This has been previously demonstrated experimentally by manipulating *Wolbachia* density using antibiotics, and by comparisons of high and low density strains [27], [44], [45], [46]. Our results strengthen this conclusion, as we show that the relationship of density and survival is strong and highly significant across a large panel of strains. Furthermore, this association does not appear to be a consequence of phylogenetic relatedness, suggesting that higher *Wolbachia* density is causing higher levels of resistance to viruses.

Do any factors other than *Wolbachia* density cause between-strain variation in the level of resistance to viruses? Our analysis provides only weak support for other factors being important, as while the genetic correlation (the proportion of genetic variance shared) between *Wolbachia* density and survival ranges from 0.57–0.76, the upper confidence intervals for all estimates are greater than 0.86. Therefore, while our data suggests other factors are important, the evidence is not strong. Any of these *Wolbachia* strains may have the intrinsic ability to provide resistance to viruses – they simply need to be present at a sufficiently high density. If true, it is tempting to speculate what this might imply about the underlying mechanism of resistance. It seems more compatible with a mechanism whereby the presence of *Wolbachia per se* makes cells or the host less hospitable to viruses, such as through competition for resources [75] or remodeling of the cellular environment. In contrast, if *Wolbachia* was expressing specific antiviral factors, then these might be easily gained or lost through evolution, breaking the genetic correlation of resistance and *Wolbachia* density. It is likely that various mechanisms can lead to variation in bacterial density and thus affect within-host density. For example, it was found recently that a ∼21 kb region encoding eight genes is amplified three to seven times in different *w*MelPop isolates relative to *w*MelCS [45], [76], and is associated with much higher density and stronger protection against viruses [45]. However, copy number variation of this region does not explain differences in density or protection between *w*MelCS and *w*Mel-like strains [45]. It is therefore tempting to speculate that genomic differences between *Wolbachia* strains that confer differential protection to viruses will only reveal different ways of varying the bacterial density rather than the actual antiviral mechanisms.

One way of rendering the host less hospitable for viruses is through the regulation of host genes. It was argued in the past that *Wolbachia* infection may lead to the activation of immune pathways that in turn could limit the multiplication of other parasites. Previous studies in mosquitoes showed that even if *Wolbachia* can prime the host immune system and increase antiviral resistance, such an effect is absent in *D. melanogaster* [44], [50], [51], [55], and flies deficient in both the Toll and IMD pathways still display *Wolbachia*-mediated resistance [54]. In agreement with previous studies, our results support the conclusion that Toll and IMD pathways are not required for antiviral protection since both *Drosomycin* and *Diptericin* expression level (reporters of Toll and IMD pathways respectively) were uncorrelated to the survival of virus-infected flies. However, other immune pathways and restriction factors could still be involved.

In the mosquito *Ae. aegypti*, the methyltransferase *AaDnmt2*, whose homolog in *Drosophila* methylates transfer RNAs and other nucleic acids, has been proposed as a potential candidate to explain the antiviral effect of *Wolbachia* [57]. *Wolbachia* was shown to decrease the expression of *AaDnmt2* through the induction of the expression of aae-miR-2940 microRNA. Conversely, the overexpression of *AaDnmt2* led to a decrease in *Wolbachia* density and an increase in the titer of dengue virus. However, it was recently shown that the *Drosophila* homolog *Dnmt2* has an antiviral effect against DCV and Nora virus, the opposite to the pattern seen in mosquito cells infected with dengue virus [59]. We found that *Wolbachia* has no consistent effect on *Dnmt2* expression in *D. simulans*, and variation in *Dnmt2* expression does not explain any of the variation in survival after infection. This suggests changes to *Dnmt2* expression are not a general explanation of the antiviral effects of *Wolbachia*. It is possible that a different mechanism of resistance applies to mosquitoes and dengue virus. However, we would argue that the critical experiment to reach this conclusion would be to show that the antiviral effects of *Wolbachia* on dengue virus require *AaDnmt2* or aae-miR-2940, and this experiment has yet to be performed.

Together with previous studies, our results show that antiviral protection is very common among *Wolbachia* strains. As such, it has to be taken into account if we are to draw a complete picture of *Wolbachia* ecology and evolution. For example, protection may favor the rapid sweeps of *Wolbachia* observed in natural populations [77], [78] and explain why strains such as *w*Mel and *w*Au, that induce weak or no CI, can be maintained in natural populations [79], [80]. Owing to the high incidence of *Wolbachia* and the broad spectrum of viruses affected by the protection, it is likely that *Wolbachia*-mediated protection has substantially contributed to the evolution of arthropods. By protecting against infection, symbiont-based immunity may in turn influence the evolution of the host immune system. Although the mechanisms remain to be elucidated, protection is tightly linked to the bacterial density. Therefore, variation in the selective pressure exerted by viruses could partly explain why *Wolbachia* strains vary so much in density, and why some are found in somatic tissues whereas other are restricted to the germ cells [81]. From an applied perspective, our study extends the panel of *Wolbachia* strains that could be introduced into mosquito populations to limit the spread of arboviruses. However, for successful introduction, the choice of a strain should not only be based on the level of protection but also consider costs on host fitness and strength of CI that will affect the invasive potential of *Wolbachia*.

## Materials and Methods

### *Wolbachia* strains, *Drosophila* lines and fly rearing

The origin of the 19 *Wolbachia* strains used in this study and their original host line are listed in Error! Reference source not found. To control for host genetic effects, all *Wolbachia* strains were transferred into the *D. simulans* STCP genetic background. This line was previously obtained through six generations of brother-sister crossing [23], [82], [83]. Eight of the strains were transferred into the STCP background in previous studies (Table 1; [23], [82], [83]). Of these, the three strains naturally-infecting *D. simulans* were generated by six generations of backcrossing *Wolbachia*-infected females to STCP males, and the remaining five were transferred by microinjection (**Table 1**). We microinjected eleven more strains into the STCP line (**Table 1**). Microinjections were performed as previously described using a microcapillary needle to transfer cytoplasm of infected embryos into uninfected STCP embryos [82]. All microinjected lines were maintained in the lab for at least 10 generations before the beginning of the experiments.

Two generations before the beginning of the experiments, the *Wolbachia* infection status of the STCP lines was checked by PCR using the diagnostic primers *wsp*81F and *wsp*691R [84], and the PCR products were sequenced. For strains microinjected in this study, vertical transmission was also assessed with PCR by testing 48 offspring per strain originating from *Wolbachia*-infected mothers (data not shown). Three fly stocks, transinfected with the strains *w*Bai, *w*Bic and *w*Bor, showed imperfect vertical transmission (54%, 91% and 62% respectively). For those three strains, the presence of *Wolbachia* was checked by PCR one generation before each experiment and only offspring from infected mothers were used in the experiments. Additionally, in qPCR assays (see below), flies of those three strains that were used in the experiments were first isolated individually, their *Wolbachia* infection status was confirmed and only *Wolbachia*-infected individuals were kept and pooled in groups of 4–6 flies.

For all the experiments, flies were maintained on a cornmeal diet (agar: 1%, dextrose: 8.75%, maize: 8.75%, yeast: 2%, nipagin: 3%) at a constant temperature of 25°C with a 12-hour light/dark cycle at 70% relative humidity.

### Inference of the *Wolbachia* phylogeny

The phylogeny of the 19 *Wolbachia* strains was inferred from the partial sequences of the eight genes *16S rRNA*, *aspC*, *atpD*, *ftsZ*, *sucB*, *groEL*, *coxA* and *fbpA* previously used in Multilocus Sequence Typing studies [66], [85]. The gene sequences were either obtained from GenBank, or were sequenced using the protocol described in Paraskevopoulos *et al.* (2006). Accession numbers and the origins of the sequences are described in Table S2. Each gene was individually aligned using Mauve v2.3.1 [86] and the phylogeny was inferred using ClonalFrame v1.2 to take into account recombination between strains [65]. To check for convergence, 9 independent runs were done with 100,000 MCMC iterations after 100,000 burn-in iterations with parameter recording every 100 iterations. For the first 8 runs, a uniformly chosen coalescent tree was used as the initial tree, and for the 9^th^ run, a UPGMA tree was used. The UPGMA starting tree was compared to the eight other trees and showed a good convergence with seven of them based on the tree comparison tool implemented in ClonalFrame v1.2 [65]. Parameter estimates for the UPGMA starting tree also showed a good convergence based on the Gelman and Rubin test [87] in ClonalFrame v1.2. A consensus tree with branch support values was built from the posterior sample of the UPGMA starting tree at 50% majority-rule in MEGA 5.2 [88] and visualized in R using the ape package [89]. The consensus tree was visually compared with a tree inferred from the concatenated sequence of the eight genes using PhyML v3.1 with 500 bootstrap replicates [90] to assess the effect of recombination on the phylogenetic signal. All clades inferred from ClonalFrame were retrieved in the maximum likelihood tree. Therefore, the ClonalFrame tree with the UPGMA starting tree was used in further analyses.

### Viral isolates

Viruses were produced and titrated as in [26], with minor changes. DCV was produced and titrated in Schneider's Line 2 cells (SL-2), while FHV was titrated in Schneider *Drosophila* line 2 cells (DL2). For each infection assay, one viral aliquot was defrosted on the day of infection and diluted in Ringer's solution [91] to reach a viral concentration of 5×10^8^ TCID_50_/mL for DCV and 3.38×10^8^ TCID_50_/mL for FHV.

### Survival assay

For each fly line, 3–6 day-old female flies were collected. After being anaesthetized with CO_2_, flies were either infected with DCV, FHV, or mock-infected with Ringer's solution [91]. The inoculum was administered by stabbing flies into the left pleural suture on the thorax with a 0.15 mm diameter anodized steel needle (*Austerlitz* Insect Pins) bent ∼0.25 mm from the end (∼half of the dorsal width of the thorax), dipped into viral or Ringer's solution as in [92]. Twenty stabbed flies were placed in a vial of fly cornmeal medium and dead flies were recorded every day for 25 days after infection. Flies were transferred into fresh vials of food every 3 days.

The survival assay was replicated on six consecutive days. On each day two vials of flies from each *Wolbachia* strain were assigned to two of the three treatments. The same was done for the *Wolbachia*-free flies, except that the number of replicates was doubled to increase statistical power. The stabbing order of the fly lines as well as the sequence of treatments were randomized each day. Mortality that occurred on the day following infection was attributed to stabbing injuries and was discarded from the analyses.

### Quantitative PCR

The *Wolbachia* density, DCV and FHV titers as well as the expression of the three host genes *Drosomycin*, *Diptericin* and *Dnmt2* were measured by qPCR on a BioRad iQ5 thermocycler using primers, probes and cycle conditions listed in Table S3. *Wolbachia* density was measured on pools of 10 virus-free 3–6 day-old female flies (n = 10 pools) from which DNA was extracted using the Gentra Puregene kit (Qiagen). For viral titers and host gene expression, 3–6 day-old flies were first infected with DCV or FHV, as described above, and, 2 days after infection, 10 flies were pooled (n = 10 pools per virus), homogenized in TRIzol Reagent (Ambion) and frozen at −80°C. Total RNA was extracted using the Direct-zol-96 RNA kit (Zymo Research) by following the manufacturer's instructions, including a 15 min DNase I digestion step.

For host gene expression, total RNA was reverse-transcribed using the GoScript Reverse Transcription System (Promega) with random primers. Host gene expression and the *Wolbachia* density were measured relative to the endogenous control gene *actin 5C* (Table S3) using the SensiFAST SYBR & Fluorescein kit (Bioline).

The copy-number of viral genomic RNA was measured relative to the control gene *EF1α100E* (Table S3) in a one-step RT-qPCR reaction using the QuantiTect Virus kit (Qiagen). For each virus, both viral and fly cDNAs were amplified in a duplex reaction using virus and fly primers in association with dual-labeled (hydrolysis) fluorescent probes (Sigma) (Table S3). For each sample, two RT-qPCR reactions were carried out and the mean of these two technical replicates was used as the relative viral titer in the statistical analysis.

The efficiency of the PCR amplication was checked using a dilution series for each set of primers. The relative *Wolbachia* density and viral titers were calculated as follows: 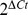, where *Ct* is the cycle threshold and 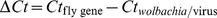.

Because qPCR efficiencies tended to be different between the control gene a*ctin 5C* and both the immune and the methyltransferase genes, we use the Pfaffl method to take into account those differences [93]. As dilution series analysis shows the qPCR efficiency for *actin 5C* to be 100%, the relative efficiency *E* for the gene of interest can be estimated from the experimental data as 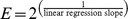. Following [93], *Ct* values for the gene of interest were corrected for differences in qPCR efficiency as 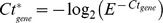. Levels of gene expression were then estimated as follow: 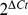, where 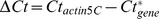. We also normalized the results across 96 well plates (sets of samples were kept in plates for both RNA extraction and qPCR). Thus, expression level for a given sample *j* was normalized by the mean, 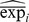, and standard deviation 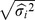 of the corresponding plate *i* for each gene of interest as follow: 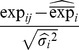. The strains *w*Bai, *w*Bic and *w*Bor were not included in the analysis of host gene expression.

### Time-course analysis of viral infection

In addition to the single timepoint analysis of viral titers, variation of titers was measured in another infection experiment for *Wolbachia*-free flies and for the *Wolbachia* strains *w*Au, *w*Sh and *w*Ana over a 5 day period. Flies were infected with DCV or FHV and maintained in the same conditions as for the other infection experiments. Live flies were frozen everyday from the day of infection until 5 dpi. For each day and each strain, the RNA was extracted from two pools of ten flies and viral titers measured as explained above except that the RT-qPCR was run on a StepOnePlus thermocycler (Applied Biosystems).

### Statistical analysis

Survival data were analyzed with a Cox's proportional hazards mixed-effect model using the coxme package in R [89]. The Cox's model estimates hazard ratios, which is the probability of a *Wolbachia-*infected fly dying at a given time-point divided by the probability of a *Wolbachia*-free fly dying. The infection treatment (DCV, FHV or mock-infected), the *Wolbachia* infection status (the 19 strains and no *Wolbachia*) were treated as fixed effects, and the replicate vial as a random effect. The overall significance of multilevel factors or their interactions was tested using likelihood ratio tests to compare models with or without these terms. Flies that were alive at the end of the experiment were treated as censored data. Variation in *Wolbachia* density and viral titers was analysed using linear models on ln-transformed data to reach the assumptions of normality and homoscedasticity. Differences in viral titer with the *Wolbachia*-free control were assessed using Dunnett's tests in order to correct for multiple comparisons.

Genetic correlations between traits were estimated by fitting a series of multi-response mixed models using a Bayesian approach in the R package MCMCglmm [94] as follows:
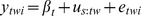
where *y_twi_* is the response of the *i^th^* biological replicate for *Wolbachia* strain *w*, for which we have measured trait *t*. *β_t_* is the intercept term for trait *t* with a level for each trait, and it can be interpreted as the mean trait value across the *Wolbachia* strains. The *Wolbachia* strain random effect, *u_s:tw_*, is the deviation from the expected value for trait *t* in strain *w*. Random effects were assumed to be from multivariate normal distributions with zero mean vectors (illustrated for a model with three traits):
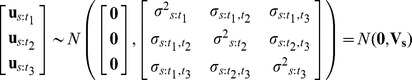
where *σ^2^_s:t1_* is the genetic variance for trait *t_1_*, and *σ_s:t1,t2_* is the genetic covariance between trait *t_1_* and *t_2_*. *e_twi_* is a residual capturing the between-vial variation for each trait (within-strain effects, environmental effects and experimental error). Residuals were assumed to be normally distributed and a separate variance was estimated for each trait with the following variance-covariance structure (illustrated for a model with three traits):
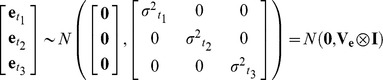
where I is an identity matrix indicating that strain effects within traits are independent of each other since traits were measured on different biological replicates.

The traits included in these models included the survival of DCV-, FHV- or mock-infected flies (estimated as a negative ln hazard ratio for each vial of flies), the *Wolbachia* density, viral titer and gene expression (all estimated as ln 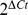). We fitted six different models with different trait combinations. Model 1 included four traits: survival after DCV-, FHV- and mock-infection, and *Wolbachia* density. Model 2 included three traits: DCV titer, FHV titer and *Wolbachia* density. Models 3 and 4 included two traits for each virus respectively: survival after viral infection and viral titer. Models 5 and 6 included four traits for each virus respectively: survival as well as *Drosomycin*, *Diptericin* and *Dnmt2* expression levels after viral infection.

Genetic correlations between two traits can arise either because the traits are causally related or because of phylogenetic non-independence. To explore these explanations we also fitted a phylogenetic mixed model, which included an additional random effect, *u_p:tw_*, which is the deviation from the expected value for trait *t* in strain *w* due to the phylogeny, i.e. the component of the between-strain variation that is explained by the phylogeny [95], [96]:
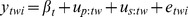
In this model the strain random effect *u_s:tw_* is the variation that is not accounted for by the phylogeny under a Brownian model of evolution [95]. The intercept *β_t_* can be interpreted as the trait value in the *Wolbachia* strain at the root of the phylogeny. For the phylogenetic effect, *u_p:tw_*, the following variance-covariance structure was assumed (illustrated for a model with three traits):
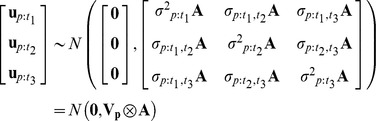
where **A** is a matrix with elements *a_jk_* standing for the proportion of time that strain *j* and *k* have had shared ancestry since the root of the phylogeny. *σ^2^_p:t1_* is the variance of the phylogenetic effect for trait *t_1_*, and *σ_p:t1,t2_* is the covariance of phylogenetic effects between trait *t_1_* and *t_2_*. Under a Brownian model of evolution, the phylogenetic covariance between two *Wolbachia* strains is inversely proportional to the time since they diverged from their common ancestor. The phylogenetic effects themselves were poorly estimated and are therefore not reported. We also fitted these phylogenetic models without the strain effect as this improved model convergence and statistical power to test for genetic correlations.

Independent normal priors with zero mean and large variance (10^10^) were used for the fixed effects and are virtually non-informative in this context. We used several prior probability distributions for the **V** *_p_* and **V** *_s_* covariance matrices to ensure our results were robust to the prior selected. Results presented were obtained using parameter expanded priors, but we also fitted models with inverse-Wishart and flat priors that gave equivalent results. We also repeated the analyses after removing outliers so that the distribution of the residuals was normal. In all cases our conclusions were unaffected by these changes. Finally, models were run after removing strains *w*Bai, *w*Bic and *w*Bor, since those strains showed unstable *Wolbachia* infection status. Estimates of genetic correlations as well as their statistical significance were very similar to models that include these 3 strains, and are therefore not reported. The models were run for 13,000,000 iterations with a burn-in of 3,000,000. We checked for convergence by visually examining the trace of the posterior sample and ensuring the autocorrelation between successive samples in the MCMC chain was <0.1. Credible intervals (CI) were estimated from the posterior distribution of parameter estimates as the 95% highest posterior density intervals.

## Supporting Information

Figure S1 **Estimated effect of** ***Wolbachia*** **on survival in mock-infected flies (Ringer's solution).** Survival is expressed as the negative natural log of the hazard ratio compared to *Wolbachia-*free flies, as estimated from a Cox's mixed-effect model. Error bars are standard errors. Symbols above the bars give the significance relative to the *Wolbachia*-free controls (*: *P*<0.05; **: *P*<0.01; ***: *P*<0.001).
(TIF)

Figure S2 **Time-course of viral titers.** (A) DCV and (B) FHV titers in *Wolbachia*-free flies (black), *w*Au- (blue), *w*Sh- (red) and *w*Ana-infected flies (light blue). Points represent the mean value of 2 replicates. Error bars are standard errors. Significance was tested using polynomial regressions with backward model selection to remove non-significant terms. For DCV, the selected model was: log2(meantiter) = μ+strain+day+day^2^+day^3^+strain×day+strain×day^2^. For FHV, the selected model was: log2(meantiter) = μ+strain+day+day^2^+strain×day+strain×day^2^. Comparisons with the *Wolbachia*-free flies showed that only *w*Au-infected flies significantly reduced viral titres, with the strain-by-day interaction (DCV: *P*<0.0001; FHV: *P*<0.0001) and the quadratic strain-by-day interaction being significant (DCV: *P*<0.0001; FHV: *P*<0.0001), indicating a slower accumulation of DCV and FHV compared to the controls.
(TIF)

Figure S3 **Immune gene and Dnmt2 expression levels after viral infection.** (A–B) Expression of *Drosomycin* after (A) DCV and (B) FHV infection. (C–D) Expression of *Diptericin* after (C) DCV and (D) FHV infection. (E–F) Expression of *Dnmt2* after (E) DCV and (F) FHV infection. Expression levels relative to the fly gene *Actin 5c* were normalised based on the qPCR plate effect (see Material and Methods). Symbols above the bars give the significance relative to the *Wolbachia*-free controls based on a Dunnett's test (*: *P*<0.05; **: *P*<0.01; ***: *P*<0.001).
(TIF)

Table S1 **Cox's proportional hazards mixed-effect model on virus- and Ringer-infected flies.** The strain-by-treatment interaction can be interpreted as the protective effect corrected for the between-strain variation in Ringer-infected flies.
(DOC)

Table S2 **MLST genes used to build the** ***Wolbachia*** **phylogeny.**
(DOC)

Table S3 **Primers and probes used in this study.**
(DOC)
