# Supplementary figures and images for: Symbionts Commonly Provide Broad Spectrum Resistance to Viruses in Insects: A Comparative Analysis of Wolbachia Strains
Source: PLoS Pathog. 2014 Sep 18;10(9):e1004369. doi: 10.1371/journal.ppat.1004369 (PMC4169468; doi:10.1371/journal.ppat.1004369)

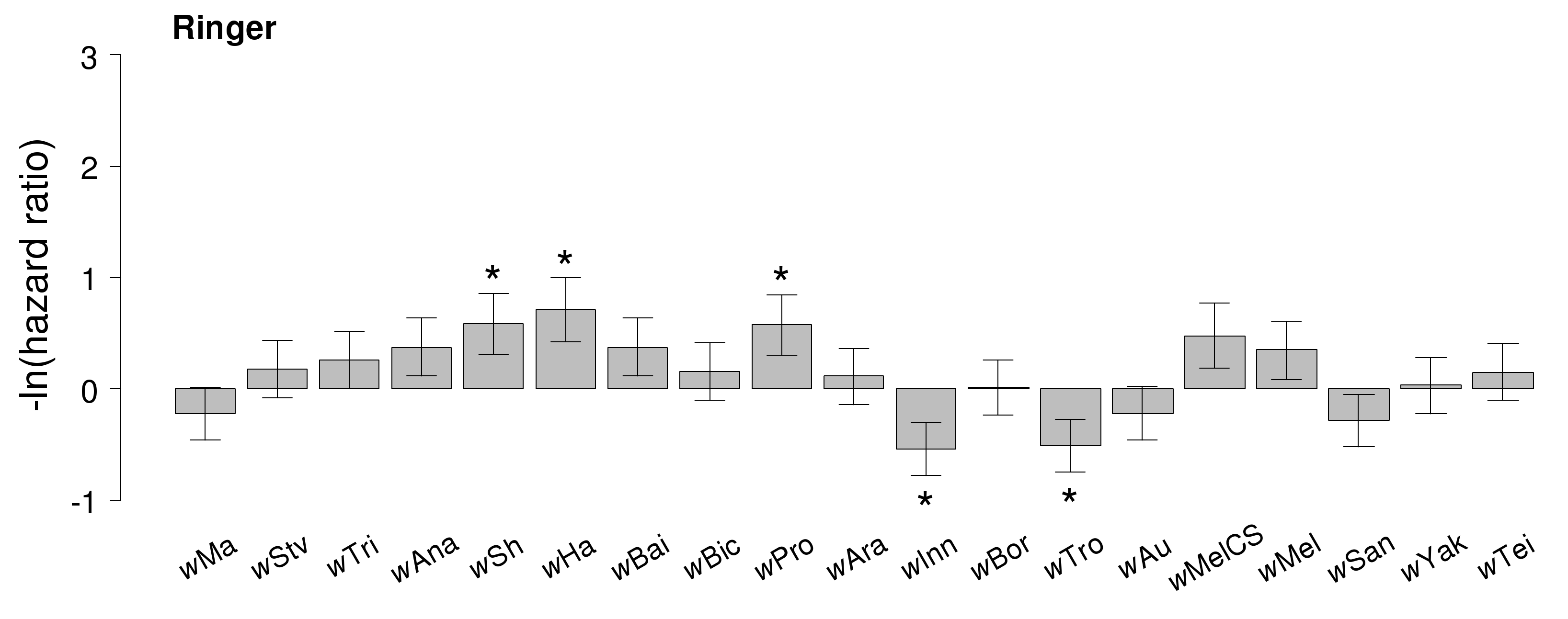

Supplement: Figure S1 — Estimated effect of Wolbachia on survival in mock-infected flies (Ringer's solution). Survival is expressed as the negative natural log of the hazard ratio compared to Wolbachia-free flies, as estimated from a Cox's mixed-effect model. Error bars are standard errors. Symbols above the bars give the significance relative to the Wolbachia-free controls (*: P<0.05; **: P<0.01; ***: P<0.001). (TIF) [file ppat.1004369.s001.tif]

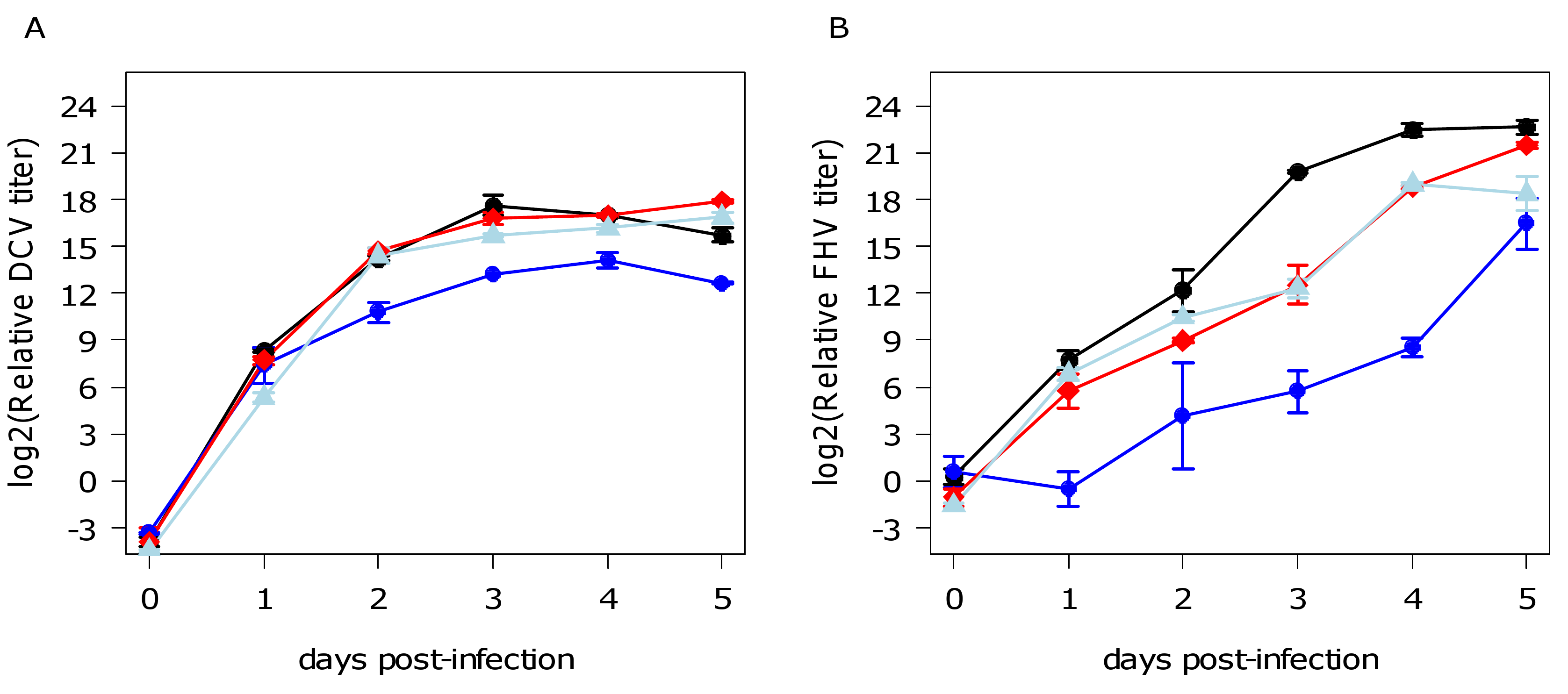

Supplement: Figure S2 — Time-course of viral titers. (A) DCV and (B) FHV titers in Wolbachia-free flies (black), wAu- (blue), wSh- (red) and wAna-infected flies (light blue). Points represent the mean value of 2 replicates. Error bars are standard errors. Significance was tested using polynomial regressions with backward model selection to remove non-significant terms. For DCV, the selected model was: log2(meantiter) = μ+strain+day+day2+day3+strain×day+strain×day2. For FHV, the selected model was: log2(meantiter) = μ+strain+day+day2+strain×day+strain×day2. Comparisons with the Wolbachia-free flies showed that only wAu-infected flies significantly reduced viral titres, with the strain-by-day interaction (DCV: P<0.0001; FHV: P<0.0001) and the quadratic strain-by-day interaction being significant (DCV: P<0.0001; FHV: P<0.0001), indicating a slower accumulation of DCV and FHV compared to the controls. (TIF) [file ppat.1004369.s002.tif]

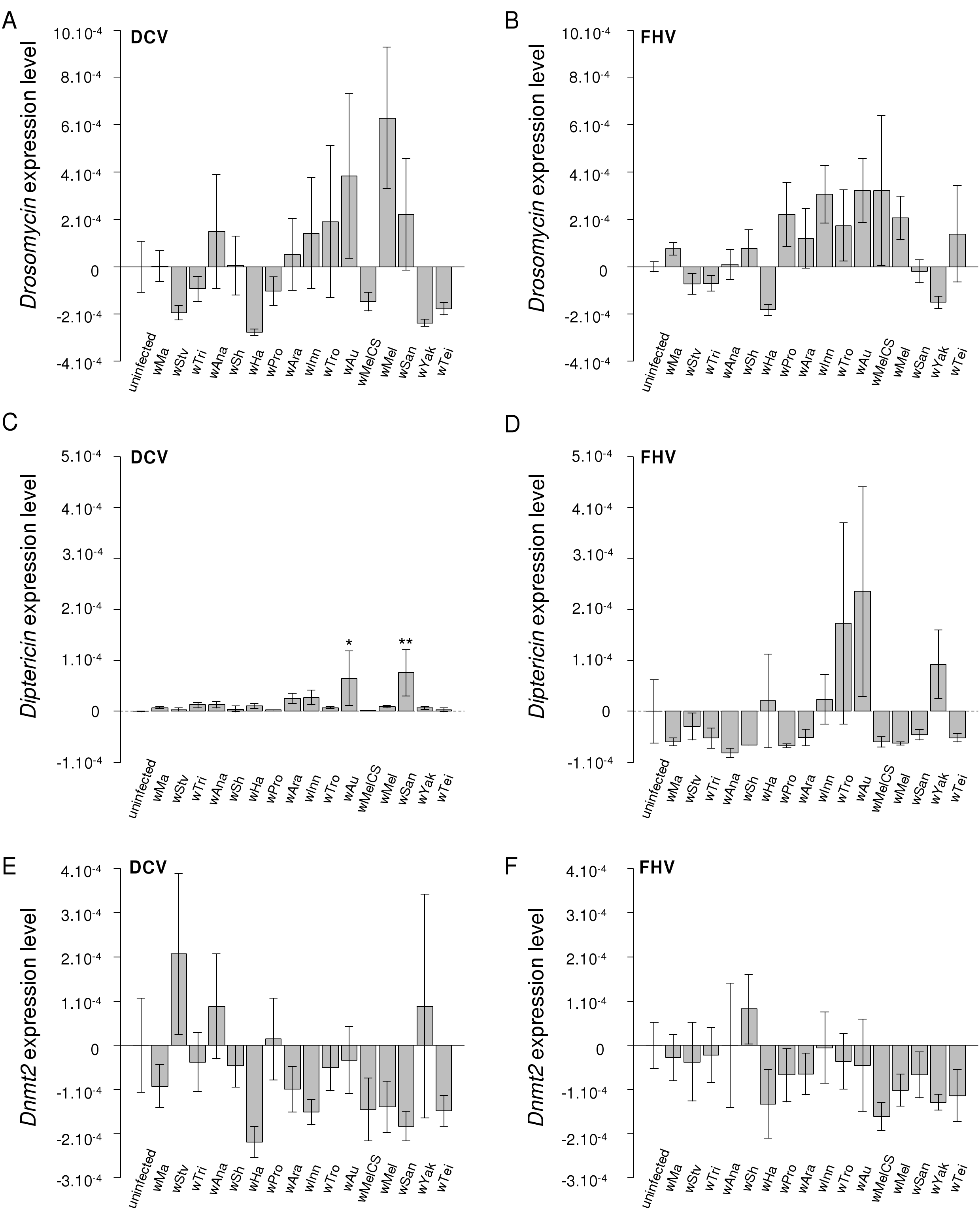

Supplement: Figure S3 — Immune gene and Dnmt2 expression levels after viral infection. (A–B) Expression of Drosomycin after (A) DCV and (B) FHV infection. (C–D) Expression of Diptericin after (C) DCV and (D) FHV infection. (E–F) Expression of Dnmt2 after (E) DCV and (F) FHV infection. Expression levels relative to the fly gene Actin 5c were normalised based on the qPCR plate effect (see Material and Methods). Symbols above the bars give the significance relative to the Wolbachia-free controls based on a Dunnett's test (*: P<0.05; **: P<0.01; ***: P<0.001). (TIF) [file ppat.1004369.s003.tif]
