# Supplementary material for: Symbionts Commonly Provide Broad Spectrum Resistance to Viruses in Insects: A Comparative Analysis of Wolbachia Strains
Source: PLoS Pathog. 2014 Sep 18;10(9):e1004369. doi: 10.1371/journal.ppat.1004369 (PMC4169468; doi:10.1371/journal.ppat.1004369)
Supplement: Table S1 — Cox's proportional hazards mixed-effect model on virus- and Ringer-infected flies. The strain-by-treatment interaction can be interpreted as the protective effect corrected for the between-strain variation in Ringer-infected flies. (DOC) [file ppat.1004369.s004.doc]

|  | DCV/Ringer-infected flies | | |  | FHV/Ringer-infected flies | | |
| --- | --- | --- | --- | --- | --- | --- | --- |
| Fixed effects | Ln(hazard ratio) | Standard error | *P*-value |  | Ln(hazard ratio) | Standard error | *P*-value |
| virus | 3.19 | 0.19 | > 0.00001 |  | 3.15 | 0.19 | > 0.00001 |
| *w*Ana | -0.38 | 0.27 | 0.17 |  | -0.37 | 0.27 | 0.16 |
| *w*Ara | -0.11 | 0.26 | 0.67 |  | -0.11 | 0.26 | 0.67 |
| *w*Au | 0.24 | 0.25 | 0.35 |  | 0.24 | 0.25 | 0.33 |
| *w*Bai | -0.38 | 0.27 | 0.16 |  | -0.38 | 0.27 | 0.15 |
| *w*Bic | -0.17 | 0.26 | 0.52 |  | -0.17 | 0.26 | 0.51 |
| *w*Bor | -0.02 | 0.25 | 0.94 |  | -0.02 | 0.25 | 0.94 |
| *w*Ha | -0.71 | 0.3 | 0.016 |  | -0.71 | 0.29 | 0.014 |
| *w*Inn | 0.57 | 0.24 | 0.02 |  | 0.58 | 0.24 | 0.014 |
| *w*Ma | 0.22 | 0.25 | 0.38 |  | 0.21 | 0.24 | 0.38 |
| *w*Mel | -0.35 | 0.27 | 0.2 |  | -0.34 | 0.27 | 0.2 |
| *w*MelCS | -0.48 | 0.3 | 0.11 |  | -0.47 | 0.3 | 0.11 |
| *w*Pro | -0.59 | 0.28 | 0.038 |  | -0.58 | 0.28 | 0.035 |
| *w*San | 0.29 | 0.25 | 0.24 |  | 0.29 | 0.24 | 0.22 |
| *w*Sh | -0.59 | 0.28 | 0.039 |  | -0.58 | 0.28 | 0.036 |
| *w*Stv | -0.18 | 0.27 | 0.5 |  | -0.18 | 0.26 | 0.48 |
| *w*Tei | -0.16 | 0.26 | 0.56 |  | -0.16 | 0.26 | 0.54 |
| *w*Tri | -0.26 | 0.27 | 0.34 |  | -0.25 | 0.26 | 0.33 |
| *w*Tro | 0.55 | 0.25 | 0.025 |  | 0.59 | 0.24 | 0.014 |
| *w*Yak | -0.03 | 0.26 | 0.91 |  | -0.03 | 0.25 | 0.91 |
| virus × *w*Ana | -0.98 | 0.35 | 0.0045 |  | 0.31 | 0.34 | 0.36 |
| virus × *w*Ara | -1.37 | 0.34 | 0.0001 |  | -1.5 | 0.33 | > 0.00001 |
| virus × *w*Au | -2.24 | 0.34 | > 0.00001 |  | -2.41 | 0.33 | > 0.00001 |
| virus × *w*Bai | 0.32 | 0.35 | 0.36 |  | 0.36 | 0.34 | 0.29 |
| virus × *w*Bic | 0.12 | 0.34 | 0.71 |  | 0.05 | 0.33 | 0.87 |
| virus × *w*Bor | -0.15 | 0.34 | 0.66 |  | -0.15 | 0.33 | 0.65 |
| virus × *w*Ha | 0.71 | 0.36 | 0.05 |  | 0.01 | 0.35 | 0.97 |
| virus × *w*Inn | -0.28 | 0.32 | 0.38 |  | -0.52 | 0.31 | 0.1 |
| virus × *w*Ma | -0.55 | 0.33 | 0.09 |  | -0.64 | 0.32 | 0.044 |
| virus × *w*Mel | -1.34 | 0.35 | 0.0001 |  | -0.82 | 0.34 | 0.015 |
| virus × *w*MelCS | -2.37 | 0.38 | > 0.00001 |  | -1.35 | 0.36 | 0.0002 |
| virus × *w*Pro | 0.01 | 0.35 | 0.98 |  | 0.25 | 0.34 | 0.47 |
| virus × *w*San | -0.29 | 0.33 | 0.37 |  | -0.16 | 0.32 | 0.61 |
| virus × *w*Sh | 0.4 | 0.36 | 0.26 |  | 0.38 | 0.35 | 0.27 |
| virus × *w*Stv | -0.98 | 0.34 | 0.004 |  | -0.2 | 0.33 | 0.54 |
| virus × *w*Tei | -0.99 | 0.34 | 0.0034 |  | -0.7 | 0.33 | 0.033 |
| virus × *w*Tri | -0.14 | 0.34 | 0.67 |  | 0.21 | 0.33 | 0.52 |
| virus × *w*Tro | -1.08 | 0.32 | 0.0008 |  | -0.89 | 0.32 | 0.0047 |
| virus × *w*Yak | -0.01 | 0.34 | 0.97 |  | -0.52 | 0.33 | 0.11 |
