# Supplementary material for: Symbionts Commonly Provide Broad Spectrum Resistance to Viruses in Insects: A Comparative Analysis of Wolbachia Strains
Source: PLoS Pathog. 2014 Sep 18;10(9):e1004369. doi: 10.1371/journal.ppat.1004369 (PMC4169468; doi:10.1371/journal.ppat.1004369)
Supplement: Table S2 — MLST genes used to build the Wolbachia phylogeny. (DOC) [file ppat.1004369.s005.doc]

| Genes | Primers [references] | Fragment size | Accession number |
| --- | --- | --- | --- |
| *16S rRNA* | 5’-TTGTAGCCTGCTATGGTATAACT -3’ [1] | 693-694 bp | DQ412084.1, KF986984*, DQ235280.1, KF986985*, KF986986*, KF986987*, DQ235279.1, DQ235283.1, DQ235289.1, DQ235275.1, DQ235276.1, KF986988*, DQ235286.1, KF986989*, KF986990*, DQ235284.1, KF986991*, KF986992*, DQ235285.1 |
|  | 5’-GAATAGGTATGATTTTCATGT -3’ [1] |  |  |
| *aspC* | aspC49_F: 5’-ATYGCTGTRACYGATAAGGYAA-3’ [2] | 972 bp | KF986993*, KF986994*, DQ235296.1*, KF986995*, KF986996*, KF986997*, DQ235295.1, DQ235299.1, DQ235305.1, DQ235291.1, DQ235292.1, KF986998*, DQ235302.1, KF986999*, KF987000*, DQ235300.1, KF987001*, KF987002*, DQ235301.1 |
|  | aspC1134R: 5’-AGARGTWGCATAAGARATTCTRA-3’ [2] |  |  |
| *atpD* | atpD242F: 5’-ATAYAGTKCGTTGTATTGCTATG-3’ [2] | 929-930 bp | KF987003*, KF987004*, DQ235312.1, KF987005*, KF987006*, KF987007*, DQ235311.1, DQ235315.1, DQ235321.1, DQ235307.1, DQ235308.1, KF987008*, DQ235318.1, KF987009*, KF987010*, DQ235316.1, KF987011*, KF987012*, DQ235317.1 |
|  | atpD1210R: 5’-CWTCAGAYAGYTCATCCATAC-3’ [2] |  |  |
| *ftsZ* | fts1: 5’-GTATGCCGATTGCAGAGCTTG-3’ [3] | 641-650 bp | KF987043*, KF987044*, DQ235342.1, KF987045*, KF987046*, FJ415469.1, DQ235341.1, DQ235345.1, AY508999.1, DQ235339.1, KF987047*, KF987048*, DQ235348.1, KF987049*, KF987050*, DQ235346.1, F987051, KF987052*, DQ235347.1 |
|  | fts2: 5’-GCCATGAGTATTCACTTGGCT-3’ [3] |  |  |
| *sucB* | sucB358F: 5’-AAAGGRACTGGYATGGGARG-3’ [2] | 540 bp | KF987065*, KF987066*, DQ235396.1, KF987067*, KF987068*, KF987069*, DQ235395.1, DQ235399.1, DQ235405.1, DQ235391.1, DQ235392.1, KF987070*, DQ235402.1, KF987071*, KF987072*, DQ235400.1, KF987073*, KF987074*, DQ235401.1 |
|  | sucB981R: 5’-TGHGGAGGRTTWATWATCGG-3’ [2] |  |  |
| *groEL* | WgroF1: 5’-GGTGAGCAGTTGCAAGAAGC-3’ [4] | 801 bp | KF987053*, KF987054*, DQ235382.1, KF987055*, KF987056*, KF987057*, DQ235381.1, DQ235385.1, KF987058*, DQ235379.1, KF987059*, KF987060*, DQ235388.1, KF987061*, KF987062*, DQ235386.1, KF987063*, KF987064*, DQ235387.1 |
|  | WgroRev1: 5’-AGATCTTCCATCTTGATTCC-3’ [4] |  |  |
| *coxA* | coxA_F1: 5’-TTGGRGCRATYAACTTTATAG-3’ [5] | 402 bp | KF987013*, KF987014*, KF987015*, KF987016*, KF987017*, FJ415470.1, NC_021089.1, DQ842280.1, KF987018*, DQ842304.1, KF987019*, KF987020*, KF987021*, KF987022*, KF987023*, KF987024*, KF987025*, KF987026*, KF987027* |
|  | coxA_R1: 5’-CTAAAGACTTTKACRCCAGT-3’ [5] |  |  |
| *fbpA* | fbpA_F1: 5’-GCTGCTCCRCTTGGYWTGAT-3’ [5] | 429 bp | KF987028*, KF987029*, KF987030*, KF987031*, KF987032*, FJ415473.1, NC_021089.1, DQ842354.1, KF987033*, DQ842340.1, KF987034*, KF987035*, KF987036*, KF987037*, KF987038*, KF987039*, KF987040*, KF987041*, KF987042* |
|  | fbpA_R1: 5’-CCRCCAGARAAAAYYACTATTC-3’ [5] |  |  |

*this study

**References**

1. O’Neill SL, Giordano R, Colbert a M, Karr TL, Robertson HM (1992) 16S rRNA phylogenetic analysis of the bacterial endosymbionts associated with cytoplasmic incompatibility in insects. Proc Natl Acad Sci U S A 89: 2699–2702.

2. Paraskevopoulos C, Bordenstein SR, Wernegreen JJ, Werren JH, Bourtzis K (2006) Toward a Wolbachia multilocus sequence typing system: discrimination of Wolbachia strains present in Drosophila species. Curr Microbiol 53: 388–395.

3. Fukatsu T (1999) Acetone preservation : a practical technique for molecular analysis. Mol Ecol 8: 1935–1945.

4. Casiraghi M, Bordenstein SR, Baldo L, Lo N, Beninati T, et al. (2005) Phylogeny of Wolbachia pipientis based on gltA, groEL and ftsZ gene sequences: clustering of arthropod and nematode symbionts in the F supergroup, and evidence for further diversity in the Wolbachia tree. Microbiology 151: 4015–4022.

5. Baldo L, Dunning Hotopp JC, Jolley K a, Bordenstein SR, Biber S a, et al. (2006) Multilocus sequence typing system for the endosymbiont Wolbachia pipientis. Appl Environ Microbiol 72: 7098–7110.
