# Supplementary material for: Symbionts Commonly Provide Broad Spectrum Resistance to Viruses in Insects: A Comparative Analysis of Wolbachia Strains
Source: PLoS Pathog. 2014 Sep 18;10(9):e1004369. doi: 10.1371/journal.ppat.1004369 (PMC4169468; doi:10.1371/journal.ppat.1004369)
Supplement: Table S3 — Primers and probes used in this study. (DOC) [file ppat.1004369.s006.doc]

| Organism / gene | Primers and probes [references] | Product size |
| --- | --- | --- |
| DCV*^a^* | DCV_S: 5’-GACACTGCCTTTGATTAG-3’ | 135 bp |
|  | DCV_AS: 5’-CCCTCTGGGAACTAAATG-3’ |  |
|  | Probe: 5’-CACAACCGCTTCCACATATCCTG-3’ |  |
| FHV*^a^* | FHV_S: 5’-GCACAAGATTTCCGTAATG-3’ | 132 bp |
|  | FHV_AS: 5’-CGGGTTAAAGGTGTGTAA-3’ |  |
|  | Probe: 5’- CACCTGCGGATGATGCCTTC-3’ |  |
| *D. simulans* |  |  |
| *Ef1α100E^a^* | Ef1α100E_FW: 5’-ACGTCTACAAGATCGGAG-3’ | 120 bp |
|  | Ef1α100E_RV: 5’-CAGACTTTACTTCGGTGAC-3’ |  |
|  | Probe: 5’-CATCGGAACCGTACCAGTAGGT-3’ |  |
| *Actin 5C^b^* | 5’-GACGAAGAAGTTGCTGCTCTGGTTG-3’ | 193 bp |
|  | 5’-TGAGGATACCACGCTTGCTCTGC-3’ |  |
| *Drosomycin^b^* | Drosomycin_F: 5’-TACTTGTTCGCCCTCTTCG-3’ [1] | 84 bp |
|  | Drosomycin_R: 5’- GTATCTTCCGGACAGGCAGT-3’ [1] |  |
| *Diptericin^b^* | Diptericin_F2 : 5’- GGACACCAGAAGGTGTGGAC-3’ | 89 bp |
|  | Diptericin_R2 : 5’- CCATATGGTCCTCCCAAGTG-3’ |  |
| *Dnmt2^b^* | Dnmt2_F: 5’-AGCGATCGGATGCACTTACT-3’ | 98 bp |
|  | Dnmt2_R: 5’-GTGAGCTCTCGAAACCCTTG-3’ |  |
| *Wolbachia atpD^b^* | atpDQALL_F: 5’-CCTTATCTTAAAGGAGGAAA -3’ | 107 bp |
|  | atpDQALL_R: 5’-AATCCTTTATGAGCTTTTGC -3’ |  |

qPCR/RT-qPCR conditions:

^a^ 50°C for 20 min, 95°C for 5 min followed by 45 cycles of 95°C for 15 s and 60°C for 45 s.

^b^ 95°C for 2 min, followed by 45 cycles of 95°C for 5 s, 55°C for 10 s, 72°C for 5 s.

**References**

1. Tanji T, Hu X, Weber ANR, Ip YT (2007) Toll and IMD pathways synergistically activate an innate immune response in Drosophila melanogaster. Mol Cell Biol 27: 4578–4588.
